# Participant Needs, Service Utilization, and Costs in a Medicaid Housing Pilot Program

**DOI:** 10.1001/jamanetworkopen.2025.12405

**Published:** 2025-05-22

**Authors:** Monique Gill, Alyssa Craigie, Megan Holtorf, Ben Gronowski, Catherine J. Livingston

**Affiliations:** 1 Center for Outcomes Research and Education, Portland, Oregon; 2 Health Share of Oregon, Portland; 3 Oregon Health and Science University, Portland

## Abstract

**Importance:**

Housing instability and homelessness have wide-ranging health and social effects. Health systems increasingly recognize the importance of addressing these needs to make progress toward health equity goals and reduce health care costs. State Medicaid programs are uniquely positioned to support the housing needs of their members but are relatively new to the housing space and need to understand how to most effectively partner with the housing sector to improve health outcomes.

**Objective:**

To evaluate the implementation and initial outcomes of a housing benefit pilot program, including participant characteristics, needs, and service utilization.

**Design, Setting, and Participants:**

This was a cohort study involving a pilot program that provided temporary rental assistance, housing navigation, and additional supports to Medicaid members between May 2022 and October 2024. The pilot took place in the Portland, Oregon, metropolitan area involving Medicaid members of Health Share of Oregon, a large coordinated care organization. Individuals were referred to the program primarily from foster care, substance use disorder residential programs, and corrections facilities. Data were analyzed from October 2024 to March 2025.

**Exposure:**

Enrolled participants received services, including temporary rental assistance, housing navigation, and other supports, for up to 12 months, with modifications made mid-pilot due to capacity challenges.

**Main Outcomes and Measures:**

Participant demographic characteristics, baseline needs, service utilization, and costs associated with the housing benefit program.

**Results:**

Of 716 referrals, 517 individuals (mean [SD] age, 37.8 [11.1] years; 295 [57.1%] male) enrolled; including 14 American Indian or Alaskan Native individuals (2.7%), 7 Asian individuals (1.4%), 67 Black individuals (13.0%), 22 Hispanic individuals (4.3%), 6 Native Hawaiian or Pacific Islander individuals (1.2%), and 308 White individuals (59.6%); 513 individuals (99.2%) spoke English. Participants had substantial housing needs, with rent (383 individuals [74.1%]) and utility (282 individuals [54.5%]) support being the most frequently reported needs and used services. The mean (SD) cost per member per month was $2225 ($1586). Frequently reported nonhousing needs included employment (154 individuals [42.3%]), food (152 individuals [41.8%]), mental health (94 individuals [25.8%]), and transportation (86 individuals [23.6%]).

**Conclusions and Relevance:**

The findings of this cohort study provide critical insights into the types of services needed and associated costs for supporting housing as a Medicaid benefit. Significant needs were observed, emphasizing the necessity of cross-sector collaboration and clear role definitions between health care and housing organizations. The findings offer valuable lessons for the design and implementation of housing support programs under Medicaid; these insights are crucial as Oregon and other states consider similar initiatives to address housing as a health-related social need.

## Key Points

**Question:**

To what extent does a Medicaid housing benefit pilot program address the housing needs of participants, and what are the financial implications of implementing the program?

**Findings:**

In this cohort study, 517 individuals were enrolled; top needs were rent and utility support, and service utilization mirrored these needs. Overall, the mean (SD) cost per member per month was $2225 ($1586).

**Meaning:**

The findings of this study of a pilot program highlight a critical need for housing supports among Medicaid members and offer lessons for design and implementation of similar programs, including a key insight on the necessity of cross-sector collaboration between health care and housing organizations.

## Teaser

This cohort study evaluates and describes the implementation and initial outcomes of a housing benefit program in Oregon.

## Introduction

Across the US, the limited supply and high cost of housing have resulted in a housing crisis, with more than one-quarter of a million individuals experiencing unsheltered homelessness and millions more unstably housed.^1^ Housing instability and homelessness have wide-ranging health and social effects, including a higher risk of substance use, morbidity, mortality, social isolation, and disruptions in education and employment.^2,3,4,5^ Given these effects, addressing housing needs for individuals experiencing housing instability can profoundly impact health outcomes.^6^

Housing instability is also associated with higher health care utilization and costs, specifically more frequent emergency department visits and more or longer inpatient hospitalizations.^7,8,9,10^ Health systems are increasingly recognizing the importance of addressing housing to support connections to preventive care, reduce health care costs, improve health outcomes, and make progress toward health equity goals.^11,12^ As part of this growing commitment to address housing as a health-related social need (HRSN), many state Medicaid agencies have begun implementing approaches to supporting the social needs of their members.^13^ State Medicaid programs are uniquely positioned to support HRSNs; however, health care organizations in general are relatively new to the housing space and have struggled to determine their role and the most effective ways to partner with the housing sector.^12^

Current regulations allow states to pay health care organizations to support Medicaid members in accessing social support services through various methods.^14^ Some states are taking this approach a step further through the use of Medicaid Section 1115 waivers, which provide an opportunity for state Medicaid programs to test innovative approaches to increase access to care, improve health outcomes, and reduce costs.^13^ In Oregon, which currently has the nation’s third-highest rate of homelessness, the state’s most recent 1115 waiver includes HRSN services as a covered benefit; implementation of the housing component of these services began in fall 2024.^15^ Previous innovations in Oregon, including the state’s 2012 Medicaid transformation and previous 1115 waiver, also included support for HRSNs and demonstrated some success in reducing health care costs and disparities^16,17^; however, it remains to be seen how Oregon’s Medicaid ecosystem will collaborate across sectors to develop a service model and deliver housing services and how Medicaid dollars will actually be used under the new waiver. Determining the financial implications of including housing as a covered benefit is of particular interest and is currently not well understood.

In addition to state agencies, Medicaid managed care organizations can play a key role in building cross-sector partnerships with community-based organizations (CBOs) and local government agencies to help develop the infrastructure for efforts to address unmet HRSNs, like housing.^18^ This is particularly true in Oregon, where the 2012 Medicaid transformation created a new type of managed care organization, coordinated care organizations (CCOs),^19^ which use a more community-driven and collaborative model to provide care. Rather than using set capitation rates for physical, behavioral, and oral health care services, CCOs have flexibility via a global budget to coordinate across health care services and support health-related services that may reduce or replace the need for health care services.^19^ The most recent 1115 waiver provides an opportunity to use this existing infrastructure and experience supporting HRSNs to test a groundbreaking version of a housing benefit, leveraging the strengths of the existing model and attempting to create additional alignment with the housing sector.

Ahead of the statewide implementation of HRSN services under Oregon’s newest 1115 waiver, the state’s largest CCO, Health Share of Oregon (hereafter, *Health Share*), developed and implemented a housing benefit pilot program.^20^ The pilot and the upcoming statewide HRSN services use an approach similar to rapid rehousing, where temporary assistance is provided to prevent or shorten the amount of time individuals and families are homeless.^21^ Health Share partnered with the Center for Outcomes Research and Education to evaluate the housing benefit pilot program. This study seeks to better understand the types of participants served by the program, their needs, and the extent to which the program met those needs, as well as to describe initial costs associated with including housing as a covered Medicaid benefit. Findings from this study provide insights into how Medicaid benefits can be used to support housing as a part of member health and offer lessons regarding benefit design and implementation as similar efforts expand in Oregon and across the country.

## Methods

This cohort study was reviewed and approved by the Providence Health institutional review board; a waiver of authorization to review participant records was granted. This study was limited to analysis of secondary data and deemed exempt from informed consent in accordance with 45 CFR 164.512(i)(2)(ii). This report follows the Strengthening the Reporting of Observational Studies in Epidemiology (STROBE) reporting guideline for cohort studies.

### Pilot Program Description and Evolution

Planning for the housing benefit pilot began in January 2022, and the program ran for 2 years, providing temporary rental assistance, housing navigation, and additional supports (eg, utility assistance and short-term hotel/motel stays) to individuals experiencing insecure housing or homelessness and families enrolled with Health Share. Eight priority populations were initially identified that would roll out over time; these populations represented key times of transition when individuals could most benefit from expedited access to housing supports. The pilot was developed in collaboration with health system partners, counties, housing experts, and a stakeholder group, including people with lived experience of homelessness. Enrollment for individuals aging out of foster care, transitioning from a substance use disorder (SUD) residential program, or exiting the corrections system began in May 2022. In late 2022, eligibility was expanded to include individuals transitioning out of recuperative care programs and 2 specific inpatient medical settings. The program originally enrolled participants for a period of 12 months. Individuals exiting assertive community treatment programs, acute care rehabilitation, or inpatient psychiatric care were slated to become eligible later in the pilot but were not launched. Capacity challenges among housing and health care partners led to a pause in enrollment from late March through the end of May 2023. When the program reopened for enrollment in June 2023, only individuals who were at risk of homelessness rather than those experiencing chronic homelessness were eligible. Additionally, only 2 of the original priority populations remained eligible for new enrollment (individuals leaving SUD residential programs and individuals aging out of foster care), participants were enrolled for a period of 6 months, and new enrollments were limited to 10 per week. These changes were made to align the program’s specifications more closely with the newly announced Medicaid benefit’s design, in addition to addressing ongoing capacity challenges. The program closed for new enrollments on October 31, 2023.

Individuals were referred to the program by a variety of sources; frequently, the referral source was the current location of the individual (eg, individuals exiting residential treatment were typically referred by the treatment program itself). On referral, a central benefit administrator (CBA) confirmed individuals’ Medicaid eligibility and enrollment in Health Share, and they were connected to a care coordinator based in the CBA and to a housing navigator at a partnering CBO. The housing navigator and care coordinator collaborated to complete initial needs assessments and then provided ongoing support to participants. As shown in Table 1, the program provided a spectrum of housing supports, including housing navigation, hotel/motel stays, move-in support and fees, monthly rent support, utility deposits and back payments, monthly utility assistance, home remediation services, home accessibility and safety modifications, healthy home goods, and renter’s insurance. This cohort study describes participants served by the program, their needs and the extent to which the program met those needs, and the initial costs associated with including housing as a covered Medicaid service.

**Table 1.** Types of Support Provided by the Housing Benefit Pilot Program

Benefit category	Description/examples	Recommended rate or cap
Housing navigation, support, and sustaining services	Includes pre- and posttenancy support and wraparound support services. Paid to community partners as a PMPM rate.	$387.00 PMPM
Monthly rent support	Rental or mortgage assistance.	Up to 12 mo initially, later revised to up to 6 mo, no cap but recommended to be based on local rent reasonableness calculations
Utility support (reinstatements, deposits, and monthly assistance)	Includes back payments/reinstatements, deposits, and monthly assistance for gas, electric, phone/internet, garbage/waste, water/sewer.	Reinstatements, $1500; arrears, $1500; deposits, $500; monthly assistance, ≤12 mo initially, later revised to ≤6 mo
Move-in fees	Includes security deposit, pet deposit, new identification cards, and background/credit check.	1-Time payment, no cap
Move-in support	Includes living and dining furniture, bedroom furniture and items, and cleaning supplies	$1200-$2400, Depending on housing size
Renter’s insurance	Assistance with payment for renter’s insurance.	1-Time payment, no cap
Hotel or motel stays	≤28-d Stay in a hotel or motel; can be used 3 separate times.	3 Requests per lifetime of the benefit, up to $130 per night
Home accessibility or safety modifications	Includes entrance ramps, grab bars in bathtubs, pest control, items to reduce environmental toxins	$10 000 Per lifetime of benefit

Abbreviation: PMPM, per member per month.

### Data Sources and Measures

Participant demographic information, including age, gender, race, ethnicity, language preference, disability and pregnancy status, county of residence, and monthly enrollment details, was obtained through Health Share’s medical claims and member enrollment files. Participant information on gender, race, ethnicity, and language preferences are self-reported at the time of Medicaid enrollment. Race and ethnicity within the member enrollment files are then grouped by the state using 2023 Office of Management and Budget standard definitions, including American Indian or Alaskan Native, Asian, Black, Hispanic, Native Hawaiian or Pacific Islander, White, and other (including categories not available to participants during the Medicaid enrollment process). Program data were the source of information on participants’ baseline needs and service utilization. Specific program data sources included referral forms, which captured additional participant demographics and priority population; a general assessment, which was completed at enrollment and collected information on participants’ health and social needs; a housing needs assessment, which included information about members’ health, social, and housing needs at enrollment; a housing plan, which captured the plan for addressing participants’ housing needs; and invoices for benefit utilization. There was a high degree of missingness in housing needs assessments (30%) due to differences in pilot implementation across CBOs and individual staff practices, including belated implementation of the needs assessment as a required form. Additionally, only 82% of individuals with a completed housing needs assessment could be matched to Medicaid enrollment data due to data entry errors in the housing needs assessment. The housing needs assessment was administered by housing navigators; participants could describe their most recent housing type or select prefer not to respond to questions about their most recent living situation; select yes, no, or unknown to questions about their housing histories; and choose yes, no, unknown, or prefer not to respond to answer questions about their criminal, medical, substance use, and mental health histories; additionally, participants could skip any questions that they did not know the answer to or to which they did not want to respond.

Invoice expenditures were aggregated by individual and by service category, forming a longitudinal dataset. Duration of program participation was determined from individual enrollment and termination dates. Per-member per-month expenditures for housing navigation and other services, as well as mean total cost per enrolled member were estimated for those who received at least 1 service within each benefit category. A binary variable was constructed to signify whether a participant used any service within a category.

### Statistical Analysis

Participant data from all available data sources were matched using a unique member identifier. Descriptive statistics were used to describe demographic characteristics and baseline assessments of housing, health, and social needs of the participants. Housing needs assessments and invoices were used to descriptively compare participant-reported needs for services to utilization and costs. All analyses were conducted using R software version 4.3.1 (R Project for Statistical Computing). Data were analyzed from October 2024 to March 2025.

## Results

### Participant Characteristics

A total of 716 individuals were referred to the program, of whom 517 individuals (72.2%) were enrolled (mean [SD] age, 37.8 (11.1) years; 295 [57.1%] male), including 14 American Indian or Alaskan Native individuals (2.7%), 7 Asian individuals (1.4%), 67 Black individuals (13.0%), 22 Hispanic individuals (4.3%), 6 Native Hawaiian or Pacific Islander individuals (1.2%), and 308 White individuals (59.6%); most participants were English speakers (513 individuals [99.2%]) and resided in Multnomah County (348 individuals [67.3%]). Individuals referred to the program but not enrolled were either not in an eligible priority population or failed to meet other enrollment criteria (eg, not a Health Share member). Additional characteristics of participants enrolled in the housing benefit program are shown in Table 2.

**Table 2.** Housing Benefit Program Participant Demographics

Characteristic	Participants, No. (%) (N = 517)
Age, mean (SD), y	37.8 (11.1)
Gender	
Male	295 (57.1)
Female	222 (42.9)
Race and ethnicity	
American Indian or Alaskan Native	14 (2.7)
Asian	7 (1.4)
Black	67 (13.0)
Hispanic	22 (4.3)
Native Hawaiian or Pacific Islander	6 (1.2)
White	308 (59.6)
Other^a^	29 (5.6)
Unknown	64 (12.4)
English spoken language	513 (99.2)
Disability	218 (45.2)
Pregnant	37 (7.2)
County	
Multnomah	348 (67.3)
Clackamas	91 (17.6)
Washington	78 (15.1)
Population	
Substance use disorder residential	241 (47.6)
Transitioning out of corrections	102 (20.2)
Aging out of foster care	59 (11.7)
Recuperative Care Program	50 (9.9)
Project nurture	38 (7.5)
Other populations	16 (3.2)
Months enrolled, mean (SD)	9.6 (4.7)

^a^ Other includes race and ethnicity categories not available to participants during the Medicaid enrollment process.

Table 3 describes participants’ housing and other histories. Prior to enrolling in the program, many participants were most recently living in transitional housing (77 individuals [21.1%]) or a treatment center (70 individuals [19.2%]). Approximately one-third of enrollees were on a housing waiting list, and many had past property or utilities debt (65 individuals [20.1%]) or a history of eviction within the last 7 years (56 individuals [16.0%]). Most participants had previously been convicted of a crime (256 individuals [73.1%]), and nearly half of participants (155 individuals [44.8%]) had been to the emergency department or hospitalized in the past year. Most participants had a history of substance use (314 individuals [88.5%]), and 204 individuals (59.5%) had a mental health diagnosis.

**Table 3.** Housing, Health, and Criminal Histories of Housing Benefit Program Participants

History	Participants, No. (%) (n = 365)
Housing	
Evicted or asked to leave in the past 7 years	56 (16.0)
Any past property or utilities debt	65 (20.1)
Currently on any housing waiting list	96 (32.0)
Recent housing situation	
Transitional housing	77 (21.1)
Treatment center	70 (19.2)
Own a house or apartment	52 (14.2)
Rental housing, no subsidy	43 (11.8)
Living with family temporarily	30 (8.2)
Permanent subsidized housing	15 (4.1)
Living with friends temporarily	10 (2.7)
Other situation^a^	53 (14.6)
Health	
Been to the emergency department or hospitalized in the past year	155 (44.8)
Any mental health diagnoses	204 (59.5)
Substance use history	314 (88.5)
Criminal	
Convicted of a crime	256 (73.1)
Convicted of a felony	201 (60.5)
Convicted of a misdemeanor	209 (64.5)

^a^ Other recent housing situations include: rental with other subsidy, hotel or motel without voucher, place not intended for habitation, hospital, psychiatric hospital, other housing type, and other setting type.

### Baseline Needs

Needs and other characteristics at the time of enrollment are shown in Table 4. At the time of referral, nearly half of participants were living alone (204 individuals [43.6%]) and many reported low levels of social support when selecting from a multiselect list of possible supports. Participants also reported case managers (174 individuals [37.2%]), counselors (127 individuals [27.1%]), and support groups (100 individuals [21.4%]) as sources of support. Outside of housing, participants reported needing additional support with employment (154 individuals [42.3%]), food (152 individuals [4.8%]), and mental health (94 individuals [25.8%]).

**Table 4.** Characteristics of Housing Program Participants’ and Nonhousing Needs at Enrollment

Characteristic	Participants, No. (%) (N = 517)
Living with	
Alone	204 (43.6)
Children	105 (22.4)
Significant other	39 (8.3)
Friends	13 (2.8)
Family members	14 (3.0)
Other^a^	17 (3.6)
Primary support systems^b^	
Family	179 (38.2)
Case manager	174 (37.2)
Counselor	127 (27.1)
Friends	123 (26.3)
Support groups	100 (21.4)
Top additional supports needed^c^	
Employment	154 (42.3)
Food	152 (41.8)
Mental health	94 (25.8)
Stability	91 (25.0)
Transportation	86 (23.6)

^a^ Other cohabitating individuals included foster parents, parent, or spouse.

^b^ Other supports systems included children, coworkers, faith based, neighbors, none, parent, partner, shelter, significant other, social worker, spiritual leader, spouse, therapist, and 12-step group.

^c^ Other additional supports needed included childcare, General Educational Development support or certifications, health conditions, relationship issues, disability, domestic or intimate partner violence resources, identification documents required for housing, safety, legal, life skills, substance use, and parenting resources (not shown); participants could choose multiple options from a multiselect list.

### Service Utilization

As shown in Table 5, support for rent, utilities, and move-in were the most frequently reported housing needs at enrollment. Table 5 also summarizes participants’ service utilization while enrolled in the program. Almost all participants had invoices for housing navigation services (469 individuals [90.7%]). Other highly used services were monthly rent support (383 individuals [74.1%]), support with move-in fees (343 individuals [66.3%]), other move-in support (320 individuals [61.9%]), and monthly utility assistance (282 individuals [54.5%]). Notably, one-third of participants (168 individuals [32.5%]) used short-term hotel or motel stays. Participants who accessed rent support received a mean (SD) of $10 963 ($7136) in assistance overall and $966 ($529) per month. The highest rates of rent support benefit use were among those transitioning out of corrections (82 individuals [80.4%]) and out of SUD residential programs (189 individuals [78.4%]) (eTable in Supplement 1). In total, across all benefit categories, the mean (SD) expenditure per member per month was $2225 ($1586).

**Table 5.** Housing Program Participants’ Baseline Housing Needs, Service Utilization, and Expenditures

Benefit category	No. (%)			Service expenditures, mean (SD), $	
	Service needs		Received ≥1 program service (n = 517)		
	Identified at baseline (n = 365)	Identified and received (n = 299)^a^		PMPM (n = 517)^a^	Total per member (n = 517)^b^
Housing monthly rent support	351 (96.4)	264 (92.6)	383 (74.1)	966 (529)	10 963 (7136)
Monthly utility assistance	298 (81.9)	194 (78.9)	282 (54.5)	88 (70)	1047 (905)
Move-in fees	227 (62.4)	154 (81.9)	343 (66.3)	189 (216)	1821 (1608)
Housing move-in support	224 (61.5)	149 (79.7)	320 (61.9)	88 (70)	896 (502)
Renter’s insurance	106 (29.0)	16 (18.8)	40 (7.7)	11 (18)	121 (193)
Utility deposit	98 (26.9)	6 (7.9)	36 (7.0)	11 (11)	116 (100)
Short-term hotel or motel stays	59 (16.2)	48 (92.3)	168 (32.5)	998 (1013)	6902 (4660)
Reinstatement utility payment	48 (13.2)	10 (28.6)	104 (20.1)	32 (37)	404 (489)
Home accessibility and safety modifications	18 (4.9)	0	1 (0.2)	78 (NA)	544 (NA)
Housing navigation, support, and sustaining services	NA	NA	469 (90.7)	402 (238)	3829 (1787)

Abbreviations: NA, not applicable; PMPM, per member per month.

^a^ Among those who had a service need identified at baseline within the respective benefit category and who had an identifier that could be matched to service invoices.

^b^ Among those who received at least 1 service in the respective benefit category.

## Discussion

This cohort study of an innovative Medicaid housing benefit pilot program provides key information about participant characteristics, necessary program services, and expected costs. The pilot program demonstrated a clear need for additional housing supports in the region, and the substantial number of individuals deemed ineligible due to not being part of a designated priority population underscores the breadth of the housing crisis. Individuals experiencing housing instability are certainly not limited to Health Share’s members or specific priority populations, and at the same time, a single program cannot meet the volume of need that has resulted from the housing crisis. This was illustrated by the need for the pilot to pause and address capacity limitations, including limiting to 2 priority populations from the original 8. Furthermore, participants reported needing additional nonhousing social supports at high rates. Intentionally building partnerships between health and social services organizations through ecosystems of care and working together collaboratively is necessary to address participants’ complex HRSNs.^22,23^

In general, baseline housing needs of enrolled participants were aligned with the services they used. Support for rent, utilities, and move-in were the most frequently reported housing needs at enrollment, and these were also the most frequently accessed services. These highly used services underscore the level of need for housing support among Medicaid members. However, while utility assistance and rent support were highly used, the level of use was still 20 to 30 percentage points lower than reported need. For example, 82% of participants reported a need for monthly utility assistance at enrollment, but only 55% of participants used this service. On the other hand, short-term hotel and motel stays were used much more frequently than would have been anticipated based on reported need; at enrollment, 16% of participants reported needing this service, but it was used by 33% of participants. For participants whose needs assessments could be matched to invoices, service utilization among those with identified needs was also examined; monthly rent, monthly utilities, move-in fees and support, and hotel and motel stays were received by most participants who identified these needs at baseline. Alternatively, many participants who identified needs for renter’s insurance, utility deposits or reinstatements, and home modifications did not receive those services. These comparisons between need and utilization were an important takeaway for the program’s design and may have implications for other programs considering what services to include or exclude. They also highlight the importance of considering the housing context outside of the program itself; limited affordable housing supply and issues finding long-term housing placements willing to accept program participants resulted in the use of hotel and motel stays as a stopgap after transitioning out of the institution from which the individual was referred, and this happened for more individuals than had identified this resource as a need at baseline. In addition, 2 significant programmatic changes made during the pilot’s pause (adjusting eligibility guidelines to focus on individuals at risk of homelessness rather than those experiencing chronic homelessness and providing the benefit for 6 rather than 12 months) could have also contributed to differences in needs and utilization, as some of the participants originally enrolled may have had needs beyond what could have been provided by the pilot. Participant histories provide additional context that may have been relevant to levels of need; for example, while 16% of participants reported being evicted or asked to move in the past 7 years, this number may underestimate the prevalence of informal evictions that they experienced. Informal evictions happen up to 5 times more often than formal ones, emphasizing the need for a systemwide approach to prevent homelessness, which could include permanent supportive housing, transitional housing, and legal interventions.^24^

The costs associated with the pilot also represent a key finding and provide a first step toward understanding what services can and should be covered by Medicaid and the related financial implications of these services. Monthly rent support, short-term hotel and motel stays, and housing navigation services were the largest types of expenditures per month and overall. As discussed, hotel and motel stays were not necessarily identified as a baseline need, but the reality of the housing search and application process meant that participants used this component of the benefit frequently, resulting in costs nearly as high as monthly rent support. In addition, while navigation services were structured as set monthly payments, the level of interaction between participants and program staff and the types of wraparound support provided varied across CBOs. In total, across all benefit categories, the mean (SD) expenditure per member per month was $2225 ($1586). In interpreting these costs, it is important to note that they do not account for implementation and infrastructure supports that were required for getting the pilot program off the ground. Beyond understanding and sharing the costs associated with providing these kinds of services in the pilot program, a critical next question in this work will be to understand the impact of these investments. Analyses are ongoing to assess participants’ health care utilization and costs during and after program enrollment, as well as pilot effects on health, housing, and social outcomes. A study of a similar program implemented under North Carolina’s current Medicaid 1115 waiver (known as the Healthy Opportunities Pilots [HOP]) recently examined health care use and spending, and researchers found increases in spending at enrollment followed by decreasing trends, adding to the body of literature suggesting that addressing HRSNs is key to improving health and reducing health care costs overall.^25^ In terms of program implementation, the pilot described in this study was similar to North Carolina’s HOP in that HOP participants who accessed housing services also frequently used navigation and utility support, but HOP did not provide support for monthly rent (ie, the most used service in the current study).^25^ The descriptive analysis presented in this study, as well as subsequent studies analyzing impacts of this pilot and Oregon’s HRSN benefit, also contribute to our growing understanding of the HRSN landscape and will allow for comparison across efforts happening in different states, like Oregon and North Carolina.

A key issue uncovered during the pilot program’s pause was the need for the program to define its intention clearly; this meant recognizing that health care agencies are not the entities best situated to provide long-term housing, especially for individuals experiencing chronic homelessness. This is skilled work often provided by housing and homeless service entities, including continuums of care and CBOs, that have the relationships, infrastructure, and staff with lived experience to meet the complex needs of this population. That said, there is still an important role for health care agencies as partners with housing and homeless services organizations that can provide a temporary bridge and resources, especially for those at risk of losing housing during times of transition, such as exiting an SUD residential program. To do this effectively, cross-sector collaboration, communication, and true partnership are critical and will require agreement on shared goals, staff training, and infrastructure investments. In addition, efforts to engage CBOs that provide culturally specific services are paramount to ensuring equitable access and supports.

### Limitations

The pilot and its evaluation experienced several limitations. For example, the flexibility in implementation was a strength of the pilot but also meant that there was significant variability in the model. Participants’ interactions with the program and the services they used were largely dependent on the housing navigator, care coordinator, and CBO to which they were connected. Previous evaluations of similar programs have emphasized the importance of consistency in service delivery models to ensure that all participants receive high-quality services.^26^ Establishing and communicating clear eligibility guidelines and service requirements can help minimize differences in experiences across participants and support CBO partners in developing processes that are sustainable and streamlined with their own internal procedures. A limitation of the evaluation presented in this study is that it relies entirely on program data, some of which were missing or unable to be matched to administrative data sources; moreover, the reliance on program data means the voices of participants are also missing from this analysis. Primary data collection with participants, including both surveys and qualitative interviews, are ongoing as individuals exit the program, and these data will be used to understand program impacts and gain insight on program experiences directly from participants. Furthermore, while information on costs may be helpful to the development of similar programs, it should be noted that costs are reported as means across priority populations for the duration of the pilot and as such may underestimate or overestimate the costs of working with members who have higher or lower needs.

## Conclusion

Overall, this cohort study provides critical information on who was served by the housing benefit program, their needs and the extent to which the program met those needs, and the cost implications of the program, all of which provide insights into how Medicaid benefits can be used to support housing as a part of health. These findings are valuable as Oregon embarks on statewide implementation of HRSN benefits, as well as nationally as other states implement similar initiatives to support housing and social needs under Medicaid. Previous research has found evidence that housing efforts save cost and reduce burden on the health care system,^9,10^ but health care partners are also new to this space. The pilot underscored the necessity of defining clear roles and collaborative frameworks between the health care and housing sectors to effectively support populations at risk for housing instability. Overall, the housing benefit pilot offers insights into the primary housing needs of Medicaid members and mean costs for these housing supports as a Medicaid benefit. While Medicaid alone cannot solve the housing crisis, better understanding costs and level of need can inform broader applications and encourage other regions and states to effectively integrate housing supports into health care systems.

## Notes

Supplement 1. **eTable.** Housing Benefit Program Service Utilization and Expenditures by Priority Population

Supplement 2.
Data Sharing Statement
