# Supplementary material for: Participant Needs, Service Utilization, and Costs in a Medicaid Housing Pilot Program
Source: JAMA Netw Open. 2025 May 22;8(5):e2512405. doi: 10.1001/jamanetworkopen.2025.12405 (PMC12100446; doi:10.1001/jamanetworkopen.2025.12405)
Supplement: Supplement 1. — eTable. Housing Benefit Program Service Utilization and Expenditures by Priority Population [file jamanetwopen-e2512405-s001.pdf]

Supplemental Online Content

Gill M, Craigie A, Holtorf M, Gronowski B, Livingston CJ. Participant needs, service utilization, and costs in a Medicaid housing pilot program. *JAMA Netw Open*. 2025;8(5):e2512405. doi:10.1001/jamanetworkopen.2025.12405

eTable. Housing Benefit Program Service Utilization and Expenditures by Priority Population

This supplemental material has been provided by the authors to give readers additional information about their work.

© 2025 Gill M et al. JAMA Network Open.

eTable 1. Housing benefit program service utilization and expenditures by priority population

Benefit Category	Overall N = 506	Substance Use Disorder (SUD) Residential N = 241	Transitioning out of Corrections N = 102	Aging out of Foster Care N = 59	Recuperative Care Program N = 50	Project Nurture N = 38	Other Populations N = 16
**Received at Least 1 Program Service, N (%)**							
Housing Navigation, Support and Sustaining Services	458 (90.5%)	226 (93.8%)	92 (90.2%)	47 (79.7%)	44 (88.0%)	36 (94.7%)	13 (81.3%)
Housing Monthly Rent Support	372 (73.5%)	189 (78.4%)	82 (80.4%)	38 (64.4%)	32 (64.0%)	24 (63.2%)	7 (43.8%)
Move-in Fees	332 (65.6%)	163 (67.6%)	70 (68.6%)	40 (67.8%)	26 (52.0%)	26 (68.4%)	7 (43.8%)
Housing Move-in Support	313 (61.9%)	149 (61.8%)	66 (64.7%)	36 (61.0%)	29 (58.0%)	25 (65.8%)	8 (50.0%)
Monthly Utility Assistance	275 (54.3%)	118 (49.0%)	68 (66.7%)	38 (64.4%)	23 (46.0%)	22 (57.9%)	6 (37.5%)
Short-Term Hotel/Motel Stays	163 (32.2%)	51 (21.2%)	27 (26.5%)	17 (28.8%)	34 (68.0%)	22 (57.9%)	12 (75.0%)
Reinstatement Utility Payment	100 (19.8%)	43 (17.8%)	22 (21.6%)	12 (20.3%)	6 (12.0%)	14 (36.8%)	3 (18.8%)
Renter's Insurance	39 (7.7%)	20 (8.3%)	7 (6.9%)	10 (16.9%)	1 (2.0%)	1 (2.6%)	0 (0.0%)
Utility Deposit	35 (6.9%)	14 (5.8%)	5 (4.9%)	11 (18.6%)	2 (4.0%)	3 (7.9%)	0 (0.0%)
Home Accessibility and Safety Modifications	1 (0.2%)	0 (0.0%)	0 (0.0%)	1 (1.7%)	0 (0.0%)	0 (0.0%)	0 (0.0%)
**Service Expenditures (PMPM), Mean (SD)**							
Housing Navigation, Support and Sustaining Services	$361 ($257)	$405 ($320)	$355 ($155)	$226 ($169)	$367 ($161)	$323 ($180)	$314 ($198)
Housing Monthly Rent Support	$707 ($622)	$730 ($603)	$928 ($660)	$649 ($675)	$505 ($519)	$441 ($453)	$428 ($610)
Move-in Fees	$122 ($198)	$143 ($244)	$117 ($134)	$131 ($171)	$52 ($118)	$111 ($128)	$65 ($144)
Housing Move-in Support	$54 ($69)	$60 ($80)	$51 ($57)	$50 ($60)	$38 ($41)	$55 ($76)	$37 ($47)

© 2025 Gill M et al. JAMA Network Open.

Benefit Category	Overall N = 506	Substance Use Disorder (SUD) Residential N = 241	Transitioning out of Corrections N = 102	Aging out of Foster Care N = 59	Recuperative Care Program N = 50	Project Nurture N = 38	Other Populations N = 16
Monthly Utility Assistance	$47 ($68)	$42 ($66)	$65 ($77)	$60 ($66)	$25 ($44)	$59 ($76)	$17 ($26)
Short-Term Hotel/Motel Stays	$326 ($748)	$139 ($391)	$215 ($640)	$160 ($321)	$981 ($1,204)	$990 ($1,299)	$857 ($852)
Reinstatement Utility Payment	$6 ($21)	$7 ($25)	$7 ($20)	$6 ($16)	$2 ($6)	$8 ($15)	$7 ($26)
Renter's Insurance	$1 ($6)	$1 ($4)	$1 ($3)	$4 ($15)	$0 ($0)	$0 ($0)	$0 ($0)
Utility Deposit	$1 ($4)	$1 ($4)	$0 ($2)	$2 ($7)	$0 ($1)	$1 ($3)	$0 ($0)
Home Accessibility and Safety Modifications	$0 ($3)	$0 ($0)	$0 ($0)	$1 ($10)	$0 ($0)	$0 ($0)	$0 ($0)
**Service Expenditures among those with services (PMPM), Mean (SD)**							
Housing Navigation, Support and Sustaining Services	$400 ($240)	$432 ($312)	$394 ($106)	$290 ($134)	$417 ($90)	$341 ($167)	$387 ($136)
Housing Monthly Rent Support	$961 ($530)	$931 ($526)	$1,154 ($528)	$1,007 ($587)	$788 ($442)	$698 ($379)	$977 ($551)
Move-in Fees	$187 ($218)	$211 ($271)	$171 ($130)	$193 ($176)	$99 ($149)	$169 ($123)	$149 ($193)
Housing Move-in Support	$87 ($70)	$97 ($82)	$79 ($53)	$81 ($57)	$65 ($33)	$84 ($80)	$74 ($39)
Monthly Utility Assistance	$87 ($70)	$85 ($72)	$97 ($76)	$94 ($60)	$54 ($51)	$102 ($75)	$46 ($22)
Short-Term Hotel/Motel Stays	$1,013 ($1,022)	$656 ($623)	$812 ($1,044)	$554 ($376)	$1,443 ($1,211)	$1,710 ($1,297)	$1,142 ($796)
Reinstatement Utility Payment	$32 ($38)	$38 ($48)	$34 ($33)	$30 ($24)	$17 ($7)	$21 ($20)	$39 ($57)
Renter's Insurance	$12 ($19)	$9 ($11)	$9 ($8)	$21 ($32)	$1 (NA)	$2 (NA)	
Utility Deposit	$11 ($11)	$12 ($13)	$7 ($5)	$13 ($11)	$6 ($5)	$9 ($10)	
Home Accessibility and Safety Modifications	$78 (NA)			$78 (NA)			

© 2025 Gill M et al. JAMA Network Open.

Benefit Category	**Overall** N = 506	**Substance Use Disorder (SUD) Residential** N = 241	**Transitioning out of Corrections** N = 102	**Aging out of Foster Care** N = 59	**Recuperative Care Program** N = 50	**Project Nurture** N = 38	**Other Populations** N = 16
**Service Expenditures (Average Total Per Member), Mean (SD)**							
Housing Navigation, Support and Sustaining Services	$3,819 ($1,796)	$3,840 ($1,719)	$4,539 ($1,768)	$2,748 ($1,489)	$3,852 ($1,725)	$3,304 ($1,919)	$3,453 ($2,117)
Housing Monthly Rent Support	$11,008 ($7,207)	$10,230 ($7,086)	$14,414 ($7,602)	$10,086 ($6,806)	$9,548 ($5,622)	$8,267 ($5,625)	$13,196 ($7,628)
Move-in Fees	$1,808 ($1,627)	$1,821 ($1,708)	$2,213 ($1,780)	$1,619 ($1,239)	$935 ($1,048)	$1,769 ($1,186)	$1,892 ($2,149)
Housing Move-in Support	$894 ($506)	$939 ($520)	$940 ($527)	$740 ($452)	$766 ($386)	$853 ($501)	$960 ($590)
Monthly Utility Assistance	$1,051 ($914)	$1,019 ($929)	$1,236 ($1,033)	$1,014 ($715)	$624 ($559)	$1,281 ($1,002)	$623 ($371)
Short-Term Hotel/Motel Stays	$6,961 ($4,641)	$5,242 ($3,759)	$6,802 ($5,512)	$4,437 ($2,871)	$8,654 ($3,838)	$9,827 ($5,322)	$8,150 ($4,518)
Reinstatement Utility Payment	$414 ($496)	$490 ($627)	$472 ($448)	$301 ($245)	$210 ($124)	$253 ($231)	$520 ($731)
Renter's Insurance	$124 ($195)	$81 ($80)	$111 ($74)	$238 ($351)	$23 (NA)	$25 (NA)	
Utility Deposit	$118 ($101)	$144 ($132)	$95 ($66)	$108 ($67)	$62 ($52)	$109 ($121)	
Home Accessibility and Safety Modifications	$544 (NA)			$544 (NA)			

© 2025 Gill M et al. JAMA Network Open.
