# Supplementary material for: Participant Needs, Service Utilization, and Costs in a Medicaid Housing Pilot Program
Source: JAMA Netw Open. 2025 May 22;8(5):e2512405. doi: 10.1001/jamanetworkopen.2025.12405 (PMC12100446; doi:10.1001/jamanetworkopen.2025.12405)
Supplement: Supplement 2. — Data Sharing Statement [file jamanetwopen-e2512405-s002.pdf]

Data Sharing Statement

Gill. Participant Needs, Service Utilization, and Costs in a Medicaid Housing Pilot Program. *JAMA Netw Open*. Published May 22, 2025. doi:10.1001/jamanetworkopen.2025.12405

Data
Data available: No

Additional Information
Explanation for why data not available: Data supporting the results of this study are not available due to privacy concerns, consent considerations, and data ownership restrictions.
